# High-Yield Production in *Escherichia coli* of Fungal Immunomodulatory Protein Isolated from *Flammulina velutipes* and Its Bioactivity Assay *in Vivo*

**DOI:** 10.3390/ijms14022230

**Published:** 2013-01-24

**Authors:** Xianghui Kong, Jiechi Zhang, Xue Han, Piqi Zhang, Xiaodong Dai, Jianing Liu, Xinxin Zhang, Imshik Lee, Shenkui Liu

**Affiliations:** 1 Key Laboratory of Saline-alkali Vegetation Ecology Restoration in Oil Field (SAVER), Ministry of Education, Alkali Soil Natural Environmental Science Center (ASNESC), Northeast Forestry University, Harbin Hexing Road, Harbin 150040, China; E-Mails: kxh29@126.com (X.K.); hanxue_home@163.com (X.H.); XXZhang@nefu.edu.cn (X.Z.); 2 Institute of Microbiology, Heilongjiang Academy of Sciences, Harbin 150010, China; E-Mails: ltzjc@vip.sina.com (J.Z.); zhangpiqi@126.com (P.Z.); heiweihlj@126.com (X.D.); liujianing0609@yahoo.com.cn (J.L.); 3 School of Physics, Nankai University, Tianjin 130071, China; E-Mail: Imshik.lee@gmail.com

**Keywords:** protein expression, large-scale production, ELISA

## Abstract

A fungal immunomodulatory protein isolated from *Flammulina velutipes* (FIP-fve) has structural similarity to the variable region of the immunoglobulin heavy chain. In the present study, the recombinant bioactive FIP-fve protein with a His-tag in *N*-terminal of recombinant protein was expressed in transetta (DE3) at a high level under the optimized culturing conditions of 0.2 mM IPTG and 28 °C. The efficiency of the purification was improved with additional ultrasonication to the process of lysozyme lysis. The yield of the bioactive FIP-fve protein with 97.1% purity reached 29.1 mg/L with a large quantity for industrial applications. Enzyme-linked immunosorbent assay showed a maximum increase in interleukin-2 (IL-2) and gamma interferon (IFN-γ) for the mice serum group of 5 mg/kg body mass (*p* < 0.01) with three doses of His-FIP-fve. However, the production of IL-4 had no apparent difference compared to the control.

## 1. Introduction

The first fungal immunomodulatory protein (FIP) was isolated from *Ganoderma lucidum* named LZ-8 or FIP-glu [1]. Since then, another six FIPs have been discovered in recent years with a primary homologous sequence of 60% to 70%, including FIP-fve from *Flammulina velutipes* [2], FIP-gts from *G. tsugae* [3], FIP-vvo from *Volvariella volvacea* [4], FIP-gja from *G. japoncium* (GenBank: AY987805), FIP-gmi from *G. microsporum* [5], and FIP-gsi from *G. sinense* [6], respectively. The natural FIPs were presented in dimerization form which showed a dumb bell-shaped structure [7] and the molecular structure of FIP is similar to a heavy-chain variable region of immunoglobulin (Ig) [7]. Ig is a group of proteins mainly existing in blood, tissue, and exocrine secretions that are responsible for the humoral immunity of mammals [8,9]. Ig plays an essential role in the body’s immune response. Given FIPs’ similar chain structure with Ig [10], FIPs are capable of inhibiting allergic reactions [1,2,11,12], promoting cytokines syntheses [13–16], activating the immune system [2,11,17], and presenting no cytotoxicity *in vitro* [15,18] and so on. As a small-molecular-weight protein, FIPs have the advantage of easy modification and potential use in wide-ranging industrial applications [19,20].

*Flammulina velutipes* belongs to Kingdom Fungi and is widely distributed in China, Russia, Siberia, Europe, North America, Australia, and so on [2]. According to previous experiments, FIP isolated from *F. velutipes* (FIP-fve), as a pure protein (contains 114 amino acids and the molecular weight is 12,704 Da) without carbohydrate [2], can be used to develop novel protein vaccines [14,15]. FIP-fve boosts the immune system, inhibits allergy formation [11,21], and stimulates the human peripheral blood lymphocytes to produce the cytokines IFN-γ and IL-2 [13,22,23]*in vitro*. The oral FIP-fve may produce an anti-inflammatory effect on OVA-induced airway inflammations, and FIP-fve may be an alternative therapy for allergic diseases and autoimmune disorder diseases [15,16]. Although FIP-fve affects the immune system similar to Ig, FIP-fve is extracted in extremely low levels from fruit bodies [2,15]. Hitherto, the expression of recombinant FIP-fve in *Escherichia coli* [11] yeast expression system, baculovirus system in insect cell lines [24] has been reported, however, it has not been reported that FIPs were applied to clinical trials or treatment. Hence, the study on large-scale production and activities of the product *in vivo* are still need to be carried out. Thus, in-depth studies of the recombinant *E. coli* for efficient expression have important significance and application value.

In this study, the optimum expression and purification conditions for obtaining soluble fusion protein (His-*FIP-fve*) from *E. coli* were investigated. The yield of the recombinant protein was found to reach 29.1 mg/L culture, which is a sufficiently large quantity for industrial applications. The immunoactivity of the purified His-FIP-fve was also tested *in vivo*.

## 2. Results and Discussion

### 2.1. Expression and Purification of His-FIP-Fve Fusion Protein in *E. coli*

**Figure 1:**
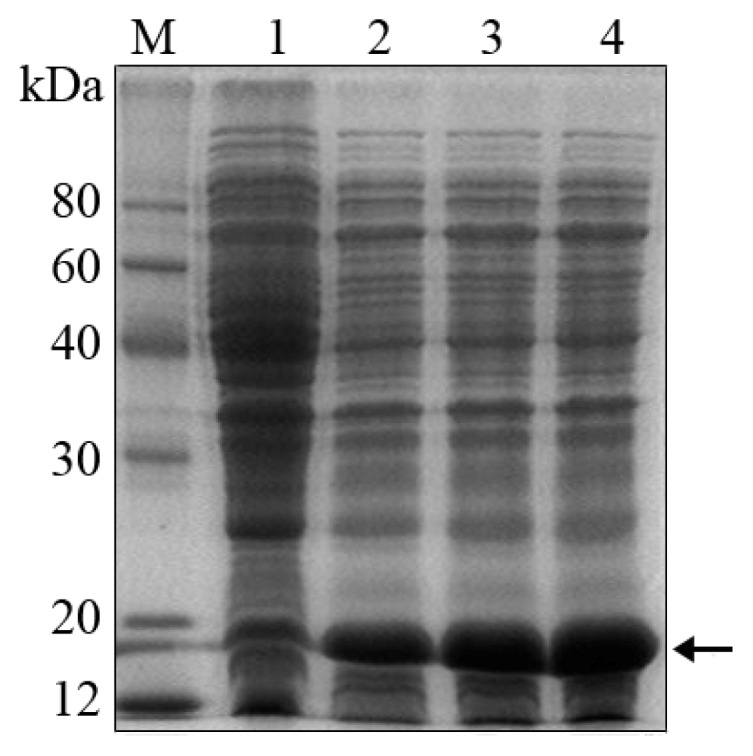
Duration of the expression of His-*FIP-fve* fusion protein. Lysates were obtained from *E. coli* (DE3) cells with pET30a-*FIP-fve* induced by 1 mM Isopropyl β-d-thiogalactoside (IPTG) and were analyzed on 12% SDS-PAGE. Lane M was a molecular weight marker. Lanes 1 to 4 were the samples induced for 0, 1.5, 3.0, and 4.5 h, respectively. The arrow indicated the target protein His-*FIP-fve*. 10 μL sample was used in SDS-PAGE after centrifugation.

To produce *FIP-fve* protein for the bioactivity assay, *FIP-fve* gene was cloned into expression vector pET30a and then *E. coli* (DE3) cells were transformed with the resulting pET30a-*FIP-fve*. Recombinant protein expression was induced with 1 mM IPTG. Lanes 1–4 showed the production of His-*FIP-fve* fusion protein for different induction time at 0, 1.5, 3.0, and 4.5 h respectively (Figure 1). The result indicated that His-*FIP-fve* fusion protein had about 18.8 kDa (arrow in Figure 1). The recombinant protein clearly increased at the induction time of 1.5 h, and the expression level of His-*FIP-fve* fusion protein increased with increased induction time from 0 h to 4.5 h.

**Figure 2:**
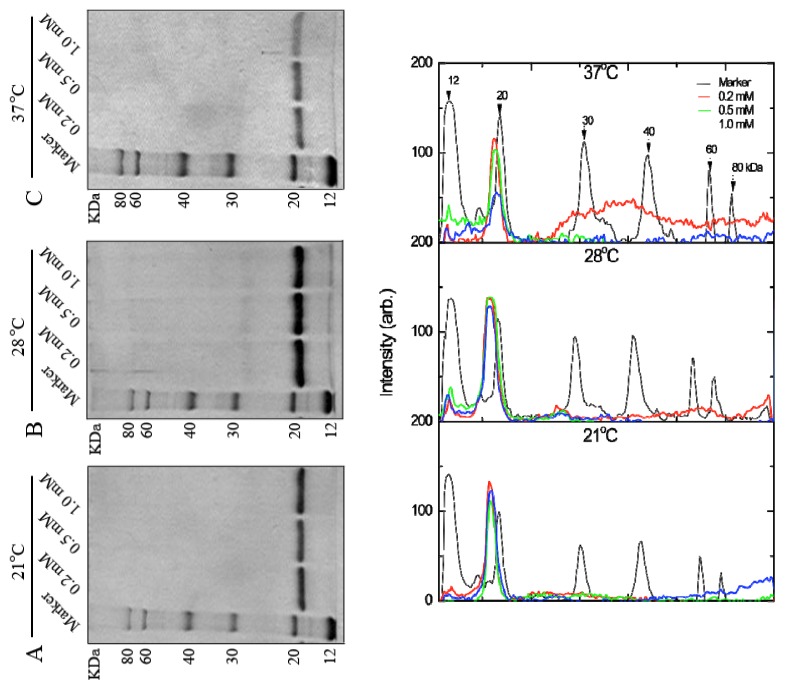
Effects of induction temperatures and IPTG concentrations on the soluble expression of His-*FIP-fve* at different temperatures: (**A**) 21 °C; (**B**) 28 °C; and (**C**) 37 °C with lane 1, molecular weight marker; lane 2, 0.2 mM IPTG; lane 3, 0.5 mM IPTG; lane 4, 1.0 mM IPTG. (**D**) Plots of (**A**), (**B**), and (**C**) after digitalization using Image J (NIH, USA). The different consecutive elution fractions were mixed and then were used for SDS-PAGE gel analysis.

To produce a large amount of His-*FIP-fve* protein for industrial applications, its high expression levels were sought in *E. coli*. The effects of the IPTG concentration (0.2, 0.5, and 1.0 mM) and culture temperatures (21, 28, and 37 °C) on the bioactive protein quantity were examined. The production of soluble protein slightly decreased with increased IPTG concentration at 21 °C and 37 °C. For incubation at 28 °C, the production of His-*FIP-fve* was improved no significant effect on the IPTG concentration (Figure 2D). These results showed that 0.2–1.0 mM IPTG concentration did not significantly affect the soluble His-*FIP-fve* protein expression level (Figure 2). His-*FIP-fve* was produced within a wide temperature range (21, 28, and 37 °C); however, lower and higher temperatures were inappropriate for maximizing production, showing that the optimum temperature for the soluble protein was at 28 °C.

**Figure 3:**
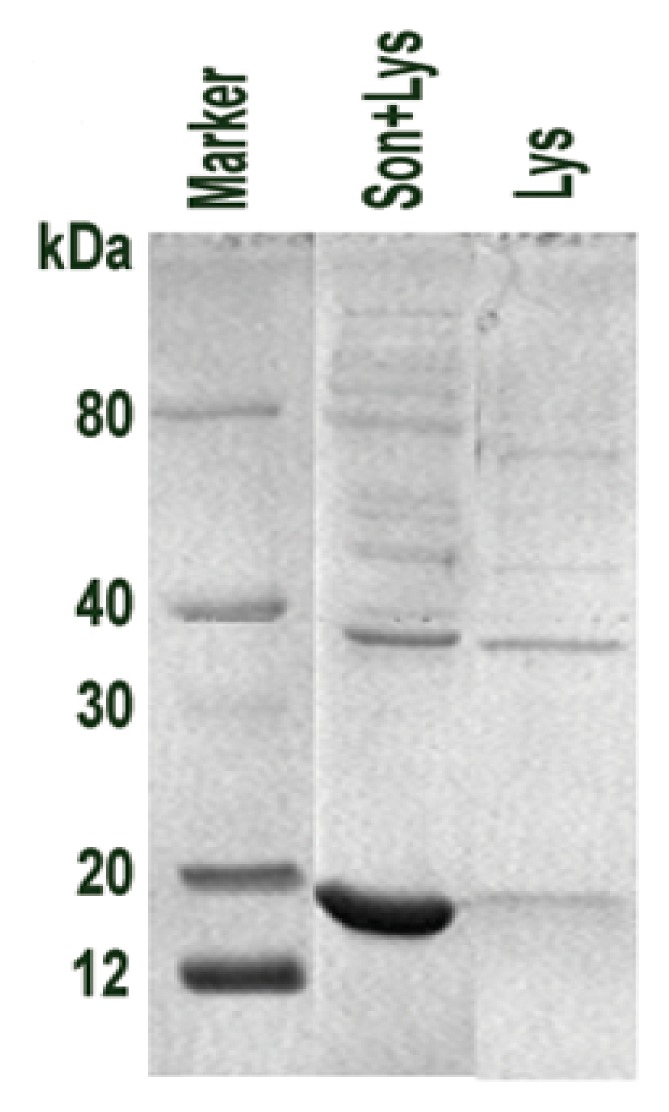
Purification optimization of His-*FIP-fve* fusion protein. The optimized lysis method of recombinant *E. coli* electrophoresed by 12% SDS-PAGE; ultrasonic lysis after lysozyme (Son+Lys) and lysozyme (Lys) methods of *E. coli* with a molecular weight marker. A sample of 3.75 μL of lysis plus 1.25 μL 4× Protein Gel Loading Buffer was used in SDS-PAGE.

To determine the optimum purification conditions, the *E. coli* lysis method was controlled to improve the lysis rate of *E. coli* and reduce the solution viscosity. Consequently, the protein yield increased. For cell lysis, the conditions for lysozyme lysis and ultrasonication were examined by microscopy and protein electrophoresis. The cell lysis rate of ultrasonication after lysozyme lysis (Figure 3, lane 1) was higher than that of lysozyme lysis (Figure 3, lane 2).

**Figure 4:**
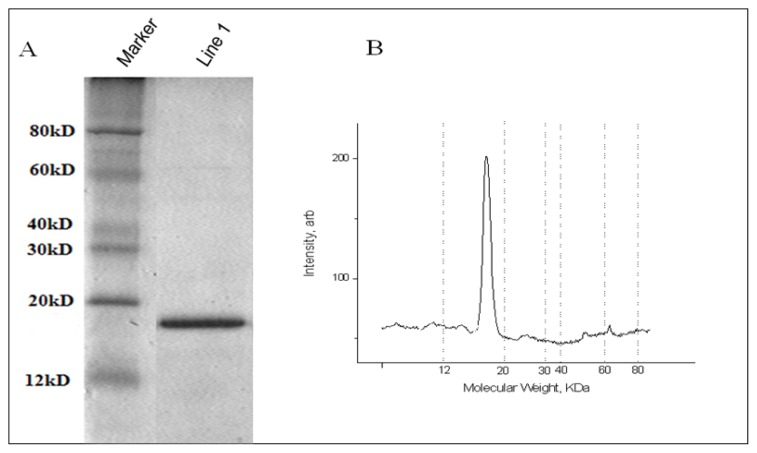
Scale-up of the expression of His-FIP-fve fusion protein. (**A**) His-FIP-fve eletrophoresised in line 1 by 15% SDS-PAGE, and (**B**) Densitometry of line 1 containing the purified His-FIP-fve fusion protein. The large peak corresponds to the purified protein representing 97.1%.

Using the optimized expression procedure, batch purification was applied to obtain a large amount of His-*FIP-fve* protein. Exactly, 5.379 g (wet weight) of cells were collected by centrifuging 1 L of cultures. Then the obtained samples were lysed by 80 mL of lysozyme buffer with additional ultrasonication. Furthermore, the obtained soluble His-*FIP-fve* proteins were purified through a His•Bind resin chromatography column with washing buffer and 30 mL of elution buffer. Subsequently, the His-*FIP-fve* protein solution was desalted by Sephadex G25 and treated by dialysis in PBS at 4 °C. Finally, 62.5 mL of His-*FIP-fve* protein solution was obtained and analyzed by Tris-Tricine SDS-PAGE (Figure 4). About 29.1 mg of His-*FIP-fve* protein (97.1% purity) obtained from 1 L of cultures was analyzed using a BCA Protein Assay Kit (Pierce) (Table 1 and Figure 4B). This large quantity of His-*FIP-fve* proteins (~5.4 mg of pure protein per gram of *E. coli* cell paste) was sufficient to analyze its bioactivities and also for industrial applications. Subsequently, we expressed and purified GST-*FIP-fve* protein, and its yield was about 5–6 mg of pure protein from 1 L of cultures with the same methods (data not shown). Compared with a GST fusion protein expression system and expression of FIP-fve mainly as inclusion bodies (insoluble) previous in *E. coli* by us [20], the *FIP-fve* recombinant proteins produced in *E. coli* reached a high quantity and soluble, which can be convenient for its future industrial applications.

Compared with other expression platforms, the *E. coli* expression system is a useful benchmark because of its advantages, such as short growth cycle and low cost [25]. Although there are some disadvantages using *E. coli* as the expression host for production of recombinant protein for research and clinical applicaton, e.g., post-translational modifications, lipopolysaccharide (LPS) contamination, yet because FIP-fve is a pure protein with a molecular weight of 12.7 kDa and without carbohydrate [2], it could be produced in *E. coli.* However, improving the expression level and effective purification are important. In this study, bioactive FIP-fve production in *E. coli* reached 29.1 mg/L, which was six times higher than the result of Ko in 1997 [11].

### 2.2. Bioactivity Assay of His-FIP-Fve

To verify whether His-*FIP-fve* possess the immunomodulatory activity of inducing cytokine secretion *in vivo*, the levels of three cytokines were measured by ELISA. Table 2 indicated the immune effects of His-FIP-fve on the serum cytokines IL-2, IL-4, and INF-γ *in vivo*. IL-2 was higher in the 5 mg/kg body mass group than in the control group (*p* < 0.01). The 5 mg/kg body mass group had a higher serum IFN-γ than in the control group (*p* < 0.01). However, for IL-4, no statistical difference was observed among all groups (*p* > 0.05). Overall, His-FIP-fve induced the production of serum IL-2 and IFN-γ, but did not change the level of IL-4. This result was similar to that for natural FIP-fve in increasing the IL-2 and IFN-γ levels *in vivo* [22], suggesting that the recombinant His-FIP-fve proteins were bioactive in terms of immunomodulatory activity.

Cytokines have an immunomodulatory function. T helper (Th) cells, including Th1 and Th2 subsets, are important immune adjustment cells. The two cell types have antagonistic functions [26]. Th1 can secrete IFN-γ, IL-2, *etc.*, and inhibit the proliferation of Th2; Th1 belongs to the cellular immune response group. Meanwhile, Th2 can secrete IL-4, *etc.*, as well as promote the proliferation and differentiation of B cells; Th2 belongs to the B cell-mediated humoral immune response group [27]. Bioactivity assay showed that the immune activity of His-*FIP-fve* may function in the stimulation of Th1 cell function by maintaining the balance between Th1 and Th2 cells and inducing the body to maintain normal immune function, which are the key points to the alleviation of disease symptoms and treatment of some diseases [28].

Previous experiments have detected the bioactivity of recombinant proteins expressed in *E. coli*, yeast, or insect cells to determine their cytokine production ability *in vitro* [24,29,30]. The methods include culturing mouse splenocytes and adding recombinant proteins *in vitro*. The test *in vitro* is a preliminary way of the recombinant protein activity. However, our experiments were performed *in vivo* by injecting His-*FIP-fve* (i.p.) based on the large amount of recombinant *FIP-fve*. The immune index would be considered accurately with both experiments *in vivo* and *in vitro*, and we are suggesting the *in vitro* invetigation for the future work.

A previous study had showed that natural *FIP-fve* stimulated the proliferation of human peripheral blood mononuclear cells (PBMCs) *in vitro* with increased transcripts of IL-2 and IFN-γ of spleen cells of mice [2], and FIP-fve stimulates interferon gamma production [13,23], and intraperitoneal (i.p.) injection of *FIP-fve* appeared to have modified immune responses in mice and have inhibitive anaphylactic responses [22]. Natural *FIP-fve* has been demonstrated to skew the response to Th1 cytokine production by oral [15,22]. Due to the complicated technology and high cost, the yield of natural *FIP-fve* was very low (87.5–165 mg/kg) [2,24], thus, selecting a suitable expression system is the premise of the application of FIP-fve. While the FIP-fve expressed in insect cells can also induce the expression of IL-2 of mouse spleen cells [24], yet it’s yield was low (6 mg/L) [25]. Meanwhile, recombinant GST-*FIP-fve* from *E. coli* with the yield (5 mg/L) only has 50% activity of natural FIP-fve [2,11], whereas we get 29.1 mg recombinant FIP-fve from 1L culture of *E. coli* Transetta (DE3), and the obtained His-FIP-fve increased the secretion of IL-2 and IFN-γ at suitable concentrations, which correlated with the findings of Wang P.H. and his colleagues in activities of nature FIP-fve in increasing the IFN-γ levels and not increasing the IL-4 levels *in vivo* [22].

## 3. Experimental Section

### 3.1. Materials

The competent cells were *E. coli* cells of Transetta (DE3) (CD801, TransGen Biotech, China). The expression vector, pET30a(+) (5422 bp), was purchased from Amersham Pharmacia Biotech.

### 3.2. Construction of Expression Plasmids

The *FIP-fve* gene-coding region (342 bp) was prepared by RT-PCR using a forward primer (5′-AGGATCCATGTCCGCCACGTCGCTCACCTT CCAG-3′; *Bam*HI site underlined) and a reverse primer (5′-GGAATTCTTAAGTCTTCTTCC ACTCAGCGA-3′; *Eco*RI site underlined). The PCR product was gel purified and then ligated into pET30a (+) at the *Bam*HI (R602A, Promega) and *Eco*RI (R6011, Promega) sites. The obtained plasmid was identified by DNA sequencing and named pET30a-*FIP-fve*.

### 3.3. Expression and Purification of His-FIP-Fve Fusion Protein

#### 3.3.1. Time Course of the Expression

The converted *E. coli* cells of DE3 cells with pET30a-FIP-fve plasmid were incubated and induced with 1 mM isopropyl β-d-thiogalactoside (IPTG; TaKaRa) upon reaching 0.8 OD_600_. The applied time course was chosen at 1.5, 3.0, and 4.5 h after IPTG induction at 30°C, meanwhile, the non-induced sample was acquired at 4.5 h. One milliliter culture was centrifugated and the pellet were suspended with 75 μL distilled water and added 4× Protein Gel Loading Buffer (R 40678, ROTRN, China), boiled at 100 °C for 5 min, and 10 μL sample was used in SDS-PAGE after centrifugation.

#### 3.3.2. Optimization Conditions

The induction temperature was controlled at 21, 28, and 37 °C with a 5 mL culture volume. The IPTG concentration was controlled at 0.2, 0.5, and 1.0 mM. To determine the optimal lysis methods of *E. coli* cells, two different lysis processes (lysozyme lysis and ultrasonic lysis) were compared. Lysozyme lysis was done under the concentration of 0.5 mg/mL lysozyme. To obtain 80 mL of cell lysis solution, the ultrasonic waves should be powered at 200 W every 5 s for 20 min on the ice.

#### 3.3.3. Large Scale Purification

After optimizing the purification condition, 1 L of cultured cells was collected by centrifugation. The obtained cells were lysed by lysozyme and ultrasonication, and then centrifuged. The samples were purified through a His•Bind resin chromatography column with binding buffer (500 mM NaCl, 20 mM Tris-HCl, and 10mM imidazole; pH 7.9), washing buffer (500 mM NaCl, 20 mM Tris-HCl, and 50 mM imidazole; pH 7.9) and with 30 mL of elution buffer (500 mM NaCl, 20 mM Tris-HCl, and 500 mM imidazole; pH 7.9). The samples were further purified by Sephadex G25 (eluted with 20 mM phosphate-buffered saline, PBS; pH 7.8) and treated by dialysis in 20 mM PBS (pH 7.8) at 4 °C.

#### 3.3.4. Electrophoresis

The protein samples were quantified using a BCA Protein Assay Kit (Pierce) and BSA as a standard sample to determine the protein content. The purified recombinant FIP-fve samples were analyzed by SDS-PAGE with molecular weight marker (80, 60, 40, 30, 20, 12 kDa, TransGen Biotect, China).

### 3.4. Bioactivity Assay of His-FIP-Fve *in Vivo*

A total of 32 Kunming strain mice (8–10 week old, 20 ± 2 g body weight) were randomly divided into four groups (*n* = 8), each group was with equal numbers of male and female. Three of them were intraperitoneally (i.p.) injected with His-FIP-fve at 5, 10, and 20 mg/kg body mass once a day for 3 days, respectively. The negative control group was treated with sterile saline. All mice were fed and maintained in specific pathogen-free conditions according to the guidelines of Care and Use of Laboratory Animals published by the China National Institute of Health. They were provided with 12 h of daylight lamp daily. About 0.8 mL blood from the inner canthus of each mouse was collected within 1 h of the last injection, dried for 2 h at room temperature, centrifuged at 3000 rpm/min for 10 min to obtain sera, and stored at −20 °C until use.

To confirm the bioactivity of His-FIP-fve, the cytokine (IFN-γ, IL-2, and IL-4) levels were detected using commercial ELISA kits, *i.e.*, # E0900004 for mouse- IFN-γ, # E0900141 for mouse IL-2, and # E0900003 for mouse IL-4 (Biosource International, Camarillo, CA, USA) according to the manufacturer’s protocol. A microtiter plate was coated with a monoclonal antibody specific for IFN-γ, IL-2 or IL-4. The cytokines found in the samples were bound by the immobilized antibody. After several washes to remove unbound proteins, an enzyme-linked polyclonal antibody which binds IFN-γ, IL-2 or IL-4 was added to the wells. The substrate solution was added after protein washing. Absorbance was measured at 450 nm using an ELISA reader (ELX800; Bio-Tek, Winooski, VT, USA). For every ELISA test, 100 μL sera sample (undiluted) was used. The concentrations of each cytokine in the samples were calculated from the corresponding standard curve and expressed as picogram per milliliter. Data were presented as the mean ± standard deviation. Statistical significance was assessed by one-way ANOVA followed by Tukey’s multiple comparison tests. *p* < 0.05 was considered as a significant difference.

## 4. Conclusions

In conclusion, we successfully expressed the His-*FIP-fve* fusion protein in *E. coli* Transetta (DE3). The high soluble protein expression conditions for His-*FIP-fve* were optimized as 0.2 mM IPTG induction at 28 °C for 24 h. After the optimized lysis method, a large amount of His-FIP-fve was purified up to 29.1 mg with 97.1% purity from 1 L of cultured cells. The obtained His-*FIP-fve* increased the secretion of IL-2 and IFN-γ at suitable concentrations *in vivo*, suggesting its bioactivity.

## Figures and Tables

**Table 1:** Summary of the purification process.

Fraction sample	Soluble protein
Wet weight of cells	5.379 g
Soluble total protein	150 mg
Purified His-FIP-fve	29.1 mg
Purity a (%)	97.1

a The purity of His-FIP-fve was assessed by densitometry analysis of Tris-Tricine SDS-PAGE. Total protein mass was estimated using a protein assay kit (Pierce) with BSA as a standard. One liter of culture media was used to purify His-Fip-fve.

**Table 2:** Effects of His-FIP-fve on the serum cytokines IL-2, IL-4, and IFN-γ in mice *in vivo* (*n* = 8). Mice were injected (i.p.) with sterile saline as negative control and His-FIP-fve by 5, 10, and 20 mg/kg body mass, and continuously treated for 3 d at 0.5 mL once a day. After 1 h during the last injection, blood was collected to obtain sera. IL-2, IL-4, and IFN-γ were detected by ELISA as described in the Materials and methods section. The levels of IL-2 (line A), IL-4 (line B), and IFN-γ (line C) were shown. *p* < 0.01 was considered an extremely significant difference and *p* < 0.05 was considered significant under the t-test. ** *p* < 0.01, * *p* < 0.05 (compared with the control).

Cytokines		Sterile saline	His-FIP-fve (mg/kg body mass)		

			5	10	20
A	IL-2 (pg/mL)	16.761 ± 2.049	20.560 ± 2.607 *	19.956 ± 3.301 *	17.662 ± 3.421
B	IL-4 (pg/mL)	37.235 ± 7.133	36.567 ± 10.156	40.675 ± 9.988	39.692 ± 9.978
C	IFN-γ (pg/mL)	10.557 ± 1.402	15.917 ± 3.872 *	12.362 ± 2.008 *	12.503 ± 1.893 *

## Abbreviations

Ig: Immunoglobin
FIP: Fungal immunomodulatory protein
FIP-fve: FIP isolated from *Flammulina velutipes*
ELISA: Enzyme-linked immunosorbent assay
IL-2: Interleukin-2
IL-4: Interleukin-4
IFN-γ: Interferon gamma
IPTG: Isopropyl β-d-thiogalactoside
